# Congenital Extrahepatic Portosystemic Shunts: Spectrum of Findings on Ultrasound, Computed Tomography, and Magnetic Resonance Imaging

**DOI:** 10.1155/2015/181958

**Published:** 2015-12-13

**Authors:** Pankaj Gupta, Anindita Sinha, Kushaljit Singh Sodhi, Anupam Lal, Uma Debi, Babu R. Thapa, Niranjan Khandelwal

**Affiliations:** ^1^Department of Radiodiagnosis and Imaging, Post Graduate Institute of Medical Education and Research (PGIMER), Chandigarh 160012, India; ^2^Pediatric Gastroenterology, Post Graduate Institute of Medical Education and Research (PGIMER), Chandigarh 160012, India

## Abstract

Congenital extrahepatic portosystemic shunt (CEPS) is a rare disorder characterised by partial or complete diversion of portomesenteric blood into systemic veins via congenital shunts. Type I is characterised by complete lack of intrahepatic portal venous blood flow due to an end to side fistula between main portal vein and the inferior vena cava. Type II on the other hand is characterised by partial preservation of portal blood supply to liver and side to side fistula between main portal vein or its branches and mesenteric, splenic, gastric, and systemic veins. The presentation of these patients is variable. Focal liver lesions, most commonly nodular regenerative hyperplasia, are an important clue to the underlying condition. This pictorial essay covers imaging characteristics in abdominopelvic region.

## 1. Introduction

Abernethy described the first case of congenital extrahepatic portosystemic shunt on autopsy on a 10-month-old female who died of unknown cause [1]. He demonstrated the absence of portal vein and existence of a mesentericocaval shunt. This is the classic description of congenital extrahepatic portosystemic shunt type I. These cases are characterised by complete absence of intrahepatic portal blood flow [2]. Type II shunts are more varied in their anatomy and are characterised by partial interruption of portal venous flow to the liver caused by portocaval, gastrorenal, mesenterico-renal, splenorenal, or mesenterico-iliac shunts [3]. The embryogenesis of this congenital anomaly is complex. Clinical presentation is variable and complex. Cases of incidental detection during imaging for evaluation of unrelated complaints are described. Adults may be diagnosed on evaluation of hepatic encephalopathy [4]. Imaging plays an important role in establishing diagnosis and detection of associated focal liver lesions and malformations that are commonly encountered in type I malformation. Biopsy is indicated in cases where findings for type I malformation are equivocal on imaging and when benign nature of the focal liver lesions cannot be established with certainty on imaging [5]. Management is guided by the type of malformation and clinical presentation. Type I malformations are not amenable to surgical or endovascular procedures. Liver transplant is the only potential therapy in patients presenting with medically recalcitrant signs and symptoms [6]. Type II malformations can be corrected by surgical ligation or endovascular occlusion [7].

## 2. Classification

**Figure 1:**
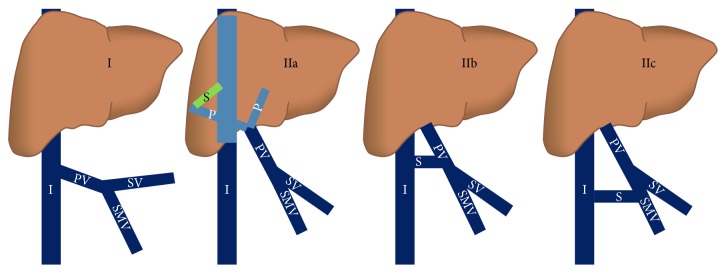
Schematic diagram showing various types of CEPS. I: IVC, P: portal vein branches, PV: portal vein, S: shunt, SMV: superior mesenteric vein, and SV: splenic vein.

Classification is based on the presence or the lack of intrahepatic portal venous flow. In type I congenital extrahepatic portosystemic shunt, there is complete shunting of the portal blood via a fistulous communication between main portal vein and inferior vena cava [2]. Intrahepatic portal venous branches are not developed. Two subtypes have been described: type Ia, where splenic vein and superior mesenteric vein drain separately into the systemic veins, and type Ib, where a splenic vein and SMV form a common channel before draining into the inferior vena cava [2, 8]. Type II congenital extrahepatic portosystemic shunt is characterised by partial diversion of the portal blood flow into the systemic veins [3]. The main portal vein may be attenuated; however, the intrahepatic portal vein branches are present. Based on the level of abnormal communication, three subtypes have been described. Type IIa shunts arise from portal vein branches and include the patent ductus venous in addition to other shunts [9]. In type IIb congenital extrahepatic portosystemic shunt, the shunts arise from the main portal vein, its bifurcation, or portomesenteric confluence. Type IIc shunts are peripheral shunts arising from gastric, mesenteric, or splenic veins. Overall, type I congenital extrahepatic portosystemic shunt is more common than type II [10]. Spontaneous closure has not been described except in patent ductus venosus [11]. Various types of shunts are depicted in Figure 1.

## 3. Embryogenesis

Congenital extrahepatic portosystemic shunt is highly complex as is the development of the portal venous system and inferior vena cava [12]. Portal vein develops from paired vitelline ducts on the anterior surface of the yolk sac. It joins primitive sinus venosus. Inferior vena cava develops from several venous channels. Hepatic segment of the inferior vena cava develops from the right end of the primitive sinus venosus. Thus, there is an embryological communication between portal vein and inferior vena cava [12].

## 4. Clinical Presentation

The clinical presentation of congenital extrahepatic portosystemic shunt is highly variable and nonspecific. There is a striking female predilection for type I congenital extrahepatic portosystemic shunt [13]. Presentation can be related to abnormal hepatic development or function: portosystemic shunt or associated congenital anomalies. Diversion of nutrient rich portal venous blood away from liver causes fatty degeneration and liver atrophy. Liver enlargement can however be noted in the presence of focal liver lesions. Most common liver masses in the setting of congenital extrahepatic portosystemic shunt are secondary to nodular regenerative hyperplasia [14]. Less commonly, focal nodular hyperplasia and hepatic adenoma may be present. The differentiation between these lesions is based on the evaluation of serum alpha-fetoprotein level, CT, and MRI. Features favouring nodular regenerative hyperplasia include multifocality, homogeneity, T1-W hyperintensity, and retention of contrast on portal venous and delayed images. Focal nodular hyperplasia and hepatic adenoma, like nodular regenerative hyperplasia, are arterial hyperenhancing lesions; however, the former characteristically shows a central T2-W hyperintense scar and the latter occurs in the setting of hormone stimulation and shows intracellular fat that can be demonstrated with chemical shift imaging. Haemorrhage is also common in hepatic adenoma and is well demonstrated with noncontrast CT and MRI. Malignant transformation in nodular regenerative hyperplasia lesions is extremely rare [15]. The basic pathogenetic mechanism for focal liver lesions is vascular derangement comprising hepatic ischemia and increased hepatic arterial flow.

Toxic metabolites bypass liver and directly enter systemic circulation in the setting of congenital extrahepatic portosystemic shunt. Toxic metabolites can result in hepatic encephalopathy, though it is rare in infants and children as the brain is relatively resistant at this age. Hepatopulmonary syndrome and digital clubbing are other manifestations in type I congenital extrahepatic portosystemic shunt. Rarely, children can present with psychiatric manifestations [8]. Serum levels of ammonia, galactose, and other toxic metabolites are elevated. Elevated galactose levels can be used for screening of neonates for CEPS. On examination, there may be liver atrophy or hepatomegaly secondary to regenerative nodules. Intermittent obstructive jaundice may be observed due to mass effect caused by regenerative nodules. Liver cirrhosis is a rare complication of CEPS type I. Ascites, splenomegaly, and varices are not a feature of CEPS.

Peripheral congenital extrahepatic portosystemic shunt can present with bleeding manifestations including vaginal or rectal bleeding [3]. Associated anomalies are consistently detected in type I congenital extrahepatic portosystemic shunt. Most common among these include cardiovascular, gastrointestinal (including polysplenia, annular pancreas, and malrotation), genitourinary, and skeletal malformations [10].

## 5. Imaging Findings

Imaging plays a crucial role in diagnosis and follow-up of patients with congenital extrahepatic portosystemic shunt.

**Figure 2:**
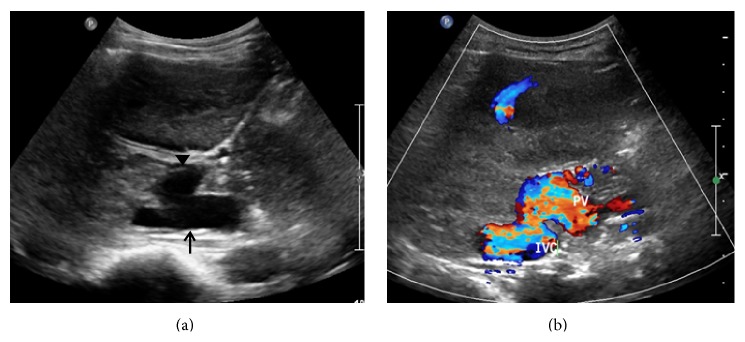
A 4-year-old boy with vague upper abdominal pain and abdominal distension since he was 2 years old. Gray-scale image (a) shows an abnormal communication between inferior vena cava (arrow) and main portal vein (arrow head). Color Doppler (b) image confirms the abnormal communication by demonstrating flow between inferior vena cava (IVC) and main portal vein (PV).

**Figure 3:**
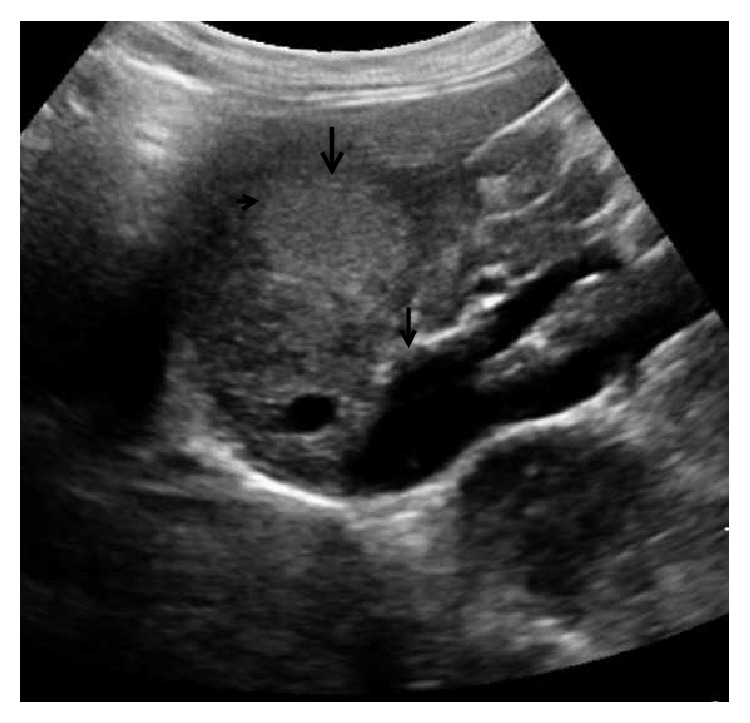
A 12-year-old female with complaints of vague upper abdominal discomfort. A well-defined hyperechoic liver lesion (arrow) with peripheral hypoechoic rim (short arrow) is seen. Thick arrow head indicates abnormal communication between main portal vein and inferior vena cava.

**Figure 4:**
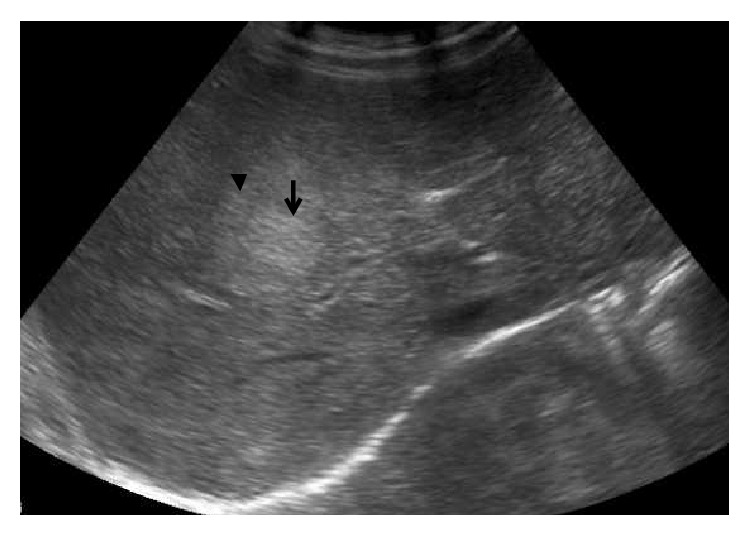
A 9-year-old boy with bleeding per rectum since he was 1 year old. A well-defined slightly hyperechoic lesion (arrow) with subtle hyperechoic rim (arrow head) is seen. This refers to carol atoll sign in nodular regenerative hyperplasia.

**Figure 5:**
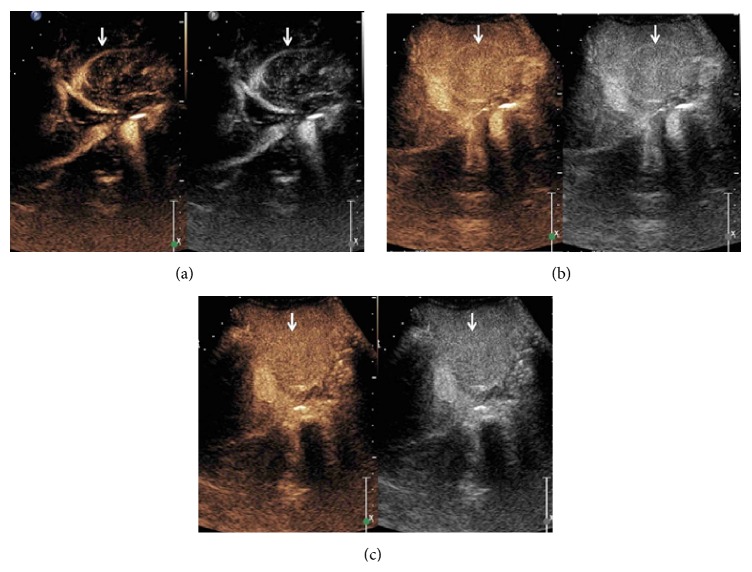
A 4-year-old boy with vague upper abdominal pain and abdominal distension since he was 2 years old. Peripheral enhancement of the lesion (a, arrow) is seen in the arterial phase of contrast enhanced US. The lesion becomes isoechoic to the adjacent liver parenchyma (b, arrow) in the venous phase of contrast enhanced US. The lesion retains contrast (c, arrow) in the delayed phase of contrast enhanced US. Points favouring nodular regenerative hyperplasia include arterial hyperenhancement and retention of contrast in the portal venous and delayed phases.

Ultrasound (US) with color Doppler is the initial imaging modality. It allows the evaluation of the shunt and liver status including liver lesions. In most cases, an absence of portal vein is detected on US in type I congenital extrahepatic portosystemic shunt. In addition, a direct fistulous communication between main portal vein and IVC may be detected (Figure 2). In type II congenital extrahepatic portosystemic shunt, the main portal vein is hypoplastic owing to the diversion of the portal blood flow. Liver size is variable and may be enlarged or atrophic. Liver echogenicity is also variable. Liver lesions in the setting of congenital extrahepatic portosystemic shunt are typically nodular regenerative hyperplasia; however, association with focal nodular hyperplasia and hepatocellular carcinoma is also known. The US appearance of these lesions is variable and may appear hyperechoic or hypoechoic (Figure 3). A characteristic finding described on gray-scale US in nodular regenerative hyperplasia is a coral atoll-like appearance. This refers to a peripheral hyperechoic rim (Figure 4) surrounding a focal liver lesion [16]. On the contrary, a halo sign, characterised by a hypoechoic rim, has also been reported (Figure 3). The role of contrast enhanced US has not been described in the setting of congenital extrahepatic portosystemic shunt. Contrast enhanced US involves intravenous administration of phospholipid shelled microbubbles (e.g., SonoVue, Bracco, Milan). Microbubbles enhance the signal of both B-mode and Doppler US. Being a blood pool agent, it does not diffuse into the interstitial spaces, unlike the iodinated contrast agent. A low mechanical index (low US power resulting in symmetrical oscillations) is utilised in general, including imaging of liver lesions. Three-phase approach studying the arterial, portal, and sinusoidal sequence is used. This parallels that employed for dynamic contrast enhanced CT or MRI. Based on the behaviour of the focal liver lesions on three phases, contrast enhanced US has been shown to accurately characterise the lesions [17]. We found contrast enhanced US useful in real-time demonstration of shunt and characterisation of liver lesions (Figures 5(a)–5(c)). In the late phase, the microbubbles are retained in the sinusoidal spaces and hence lesions containing normal hepatocytes (e.g., focal nodular hyperplasia and nodular regenerative hyperplasia) achieve similar echogenicity as the background liver parenchyma and hence disappear. However, contrast enhanced US demands an older child. The role of contrast enhanced US in congenital extrahepatic portosystemic shunt can be a subject of considerable interest for future research.

**Figure 6:**
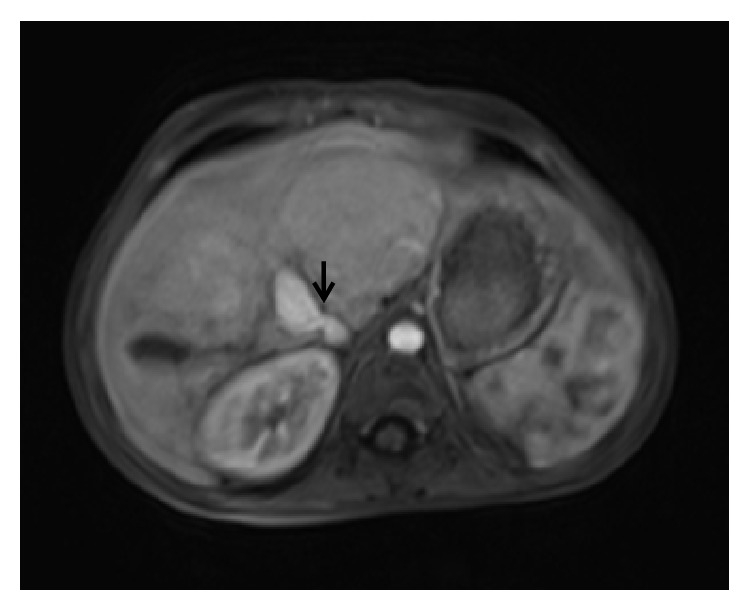
A 12-year-old female with complaints of vague upper abdominal discomfort. Axial image of MR angiography reveals an abnormal communication between main portal vein and inferior vena cava (arrow).

**Figure 7:**
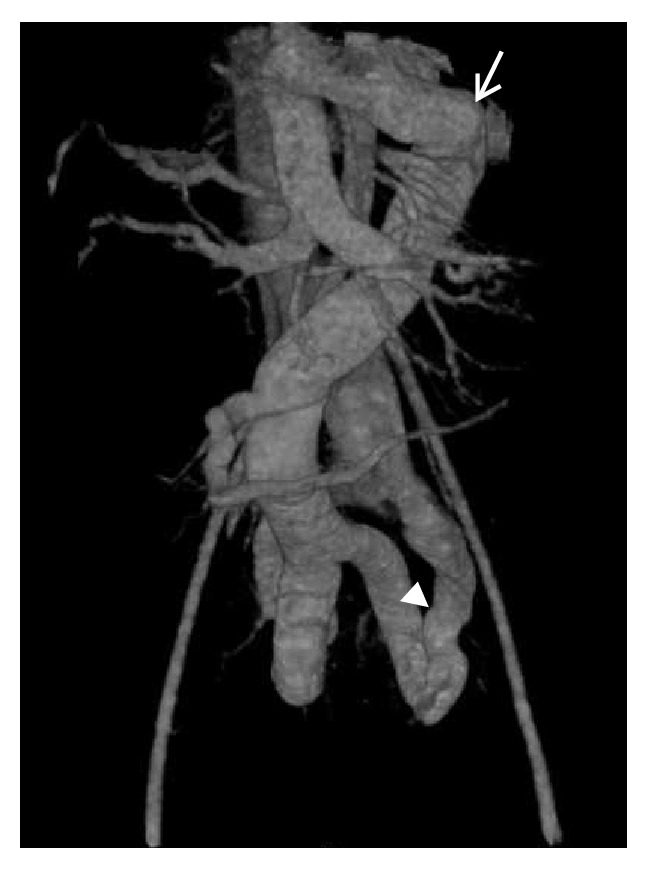
A 9-year-old boy with bleeding per rectum since he was 1 year old. Volume rendered CT image reveals dilatation of superior mesenteric vein and inferior mesenteric vein (arrow) with abnormal communication between iliac vein and branches of inferior mesenteric vein (arrow head).

**Figure 8 fig8:**
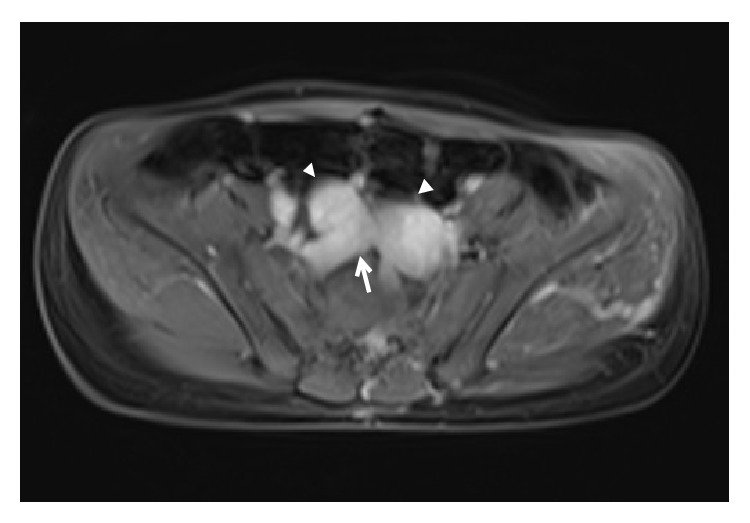
A 9-year-old boy with bleeding per rectum since he was 1 year old. Axial MR image reveals abnormal vascular channels in the pelvis suggesting a communication between tributaries of superior mesenteric vein (arrow head) and iliac veins (arrow).

**Figure 9:**
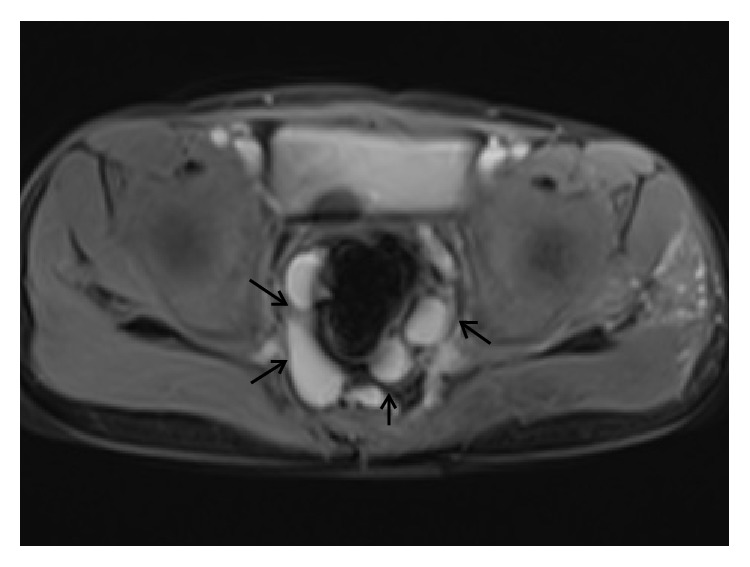
A 9-year-old boy with bleeding per rectum since he was 1 year old. Axial MR image of the same patient as above reveals abnormal perirectal vascular channels (arrows).

**Figure 10:**
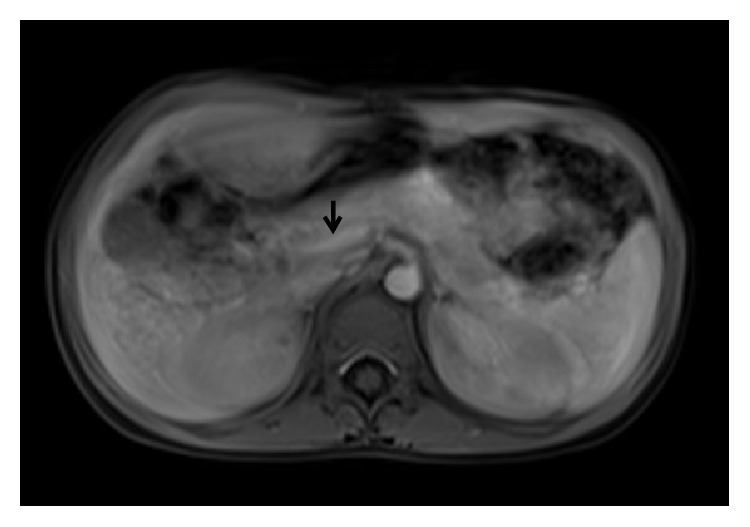
A 9-year-old boy with bleeding per rectum since he was 1 year old. Axial contrast enhanced MR image indicates hypoplastic main portal vein (arrow).

**Figure 11:**
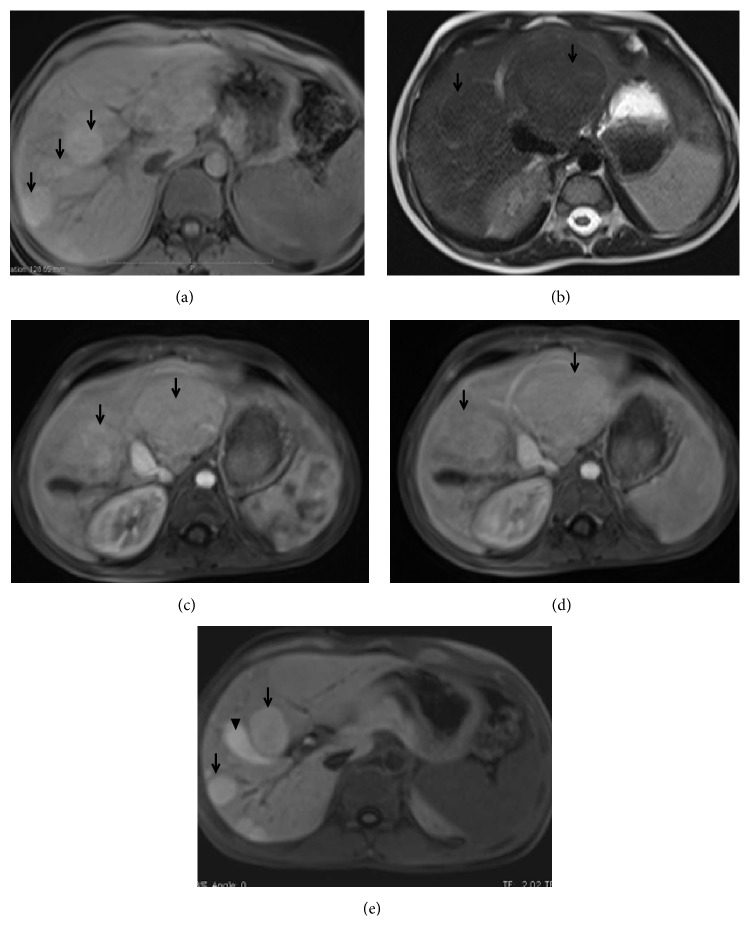
A 4-year-old boy with vague upper abdominal pain and abdominal distension since he was 2 years old. Axial T1-W image (a) shows multiple well-defined hyperintense lesions (arrows). The lesions are hypointense on T2-W images (b, arrows). Slight arterial hyperenhancement is seen with the lesions (c, arrows). There is retention of contrast in the portal venous phase (d, arrows). Hepatobiliary phase image (e) shows retention of contrast (arrows). Arrow head points to biliary excretion of contrast into gallbladder. Imaging features favouring nodular regenerative hyperplasia include multiple lesions, T1-W hyperintensity, arterial hyperenhancement, and retention of contrast on portal venous and equilibrium phases.

Diagnosis of congenital extrahepatic portosystemic shunt is confirmed by contrast enhanced MRI or CT. MRI must be preferred over CT as the latter exposes the child to ionising radiations. Besides, MRI is better for characterisation of liver lesions. Both MR angiography and CT angiography allow accurate mapping of the course of the portosystemic shunt (Figures 6 and 7). There may be nonvisualisation of intrahepatic portal vein branches; however, this does not always employ absence. Angiography (as described later) is the modality of choice for confirming the absence of portal vein branches and hence typing the shunt. Besides portosystemic shunt, shunting at other levels including mesenteric vein is also depicted well (Figures 7 [Fig fig8]–9). In type II congenital extrahepatic portosystemic shunt, portal vein is typically hypoplastic (Figure 10). Nodular regenerative hyperplasia lesions have rather characteristic appearance on MRI, allowing a noninvasive diagnosis [5]. The lesions are homogeneous and well defined and are frequently multiple. T1-W images reveal the lesions to be hyperintense (Figure 11(a)) while T2-W signal characteristics are more variable (Figure 11(b)). Most lesions are isointense to slightly hyperintense on T2-W images. The lesions show arterial hyperenhancement (Figure 11(c)) and remain isointense to slightly hyperintense on portal venous, equilibrium, and delayed phase images (Figure 11(d)). Contrast enhanced MRI adds to the diagnostic confidence in lesion characterisation and has become standard protocol in evaluation of focal liver lesions. Arterial hyperenhancement reflects the vascular supply of the nodular regenerative hyperplasia lesions from the hepatic artery. Tendency for these lesions to remain hyperintense on portal venous and delayed phases is different from other benign lesions that become isointense in these phases as well as from hepatocellular carcinoma that shows venous phase washout and appears hypointense relative to the liver parenchyma. Liver specific MRI contrast agents including gadobenate dimeglumine (MultiHance, Bracco, Milan) have unique property of hepatocyte uptake and biliary excretion. This adds to the lesion characterisation as lesions containing functioning hepatocytes are expected to retain contrast and appear isointense to the liver parenchyma on the hepatobiliary phase images. This was demonstrated in one of our patients (Figure 11(e)). The more commonly employed extracellular agents, for example, gadopentetate dimeglumine (Magnevist, Bayer, NJ), have no biliary excretion. Disadvantage of using MultiHance is the need for repeat imaging and hence repeat sedation/anaesthesia in a young child. Similar behaviour of the lesions is expected following administration of contrast in CT. Fat, calcification, and haemorrhage are not the imaging features of nodular regenerative hyperplasia.

Transrectal portal scintigraphy (with 123 I-Iodoamphetamine) is a nuclear medicine study that allows calculation of the shunt ratio in type II congenital extrahepatic portosystemic shunt [18]. This information is useful in formulating management plan in type II congenital extrahepatic portosystemic shunt.

Accurate typing of the shunt is essential for precise management. In this context, angiography should be regarded as one of the initial investigations in all patients suspected of having CEPS. Besides the transarterial portography, shunt demonstration and typing can also be achieved by direct contrast injection into the shunt with balloon occlusion. Angiography allows the measurement of portal venous pressures required for monitoring following occlusion of shunt [19].

## 6. Differential Diagnosis

Few important differential diagnoses must be considered. These include acquired portosystemic shunt, portal vein thrombosis, and intrahepatic portosystemic shunt [8]. Absence of ascites, splenomegaly, and specific collateral veins allows confident exclusion of acquired portosystemic shunt. Absence of intraluminal filling defect (in acute thrombosis) and lack of collateral veins, expansion, and wall calcification (in chronic thrombosis) rule out the possibility of portal vein thrombosis. The key to differentiation between intrahepatic portosystemic shunts and congenital extrahepatic portosystemic shunt is the location of shunt. While intrahepatic portosystemic shunts are characterised by abnormal connections between branches of the portal vein and the inferior vena cava or hepatic veins, in congenital extrahepatic portosystemic shunt, such shunting involves main portal vein or more peripheral veins.

## 7. Management

Management depends on the type of congenital extrahepatic portosystemic shunt. No definite curative surgical or endovascular therapy can be employed in type I congenital extrahepatic portosystemic shunt as the shunt is the only route for drainage of the portal blood and hence this shunt cannot be blocked. Only therapeutic option in such cases is liver transplant [6]. This treatment is reserved for patients developing features of hepatic encephalopathy. However, recently a more aggressive approach has been suggested. In a study by Blanc et al., twenty-three patients with congenital portosystemic shunts were evaluated [20]. Two patients had extrahepatic shunt; the rest have intrahepatic shunts classified on the basis of ending of the shunt in the caval system. In both patients with extrahepatic portosystemic shunts, a single stage ligation was performed. On follow-up, both the patients were alive and did not require liver transplantation. Type II shunts are amenable to surgical or endovascular treatment [7]. These therapies are guided by the shunt ratio. A shunt ratio of greater than 60% is associated with a greater risk of development of spontaneous encephalopathy. Asymptomatic patients are typically followed up clinically and with imaging studies.

## 8. Conclusion

Congenital extrahepatic portosystemic shunt should be considered clinically in children presenting with nonspecific liver dysfunction. On imaging, this vascular anomaly should be suspected when there are multiple liver lesions and lack of imaging signs of portal hypertension. Primary diagnosis can be offered with US and Doppler. MRI and CT allow classification of the shunt and evaluation of the associated congenital anomalies. Definitive management is liver transplant in type I and surgical or endovascular closure of the shunt in type II.
